# The evaluation of the effect of long-term antihypertensive drug usage on bone density with dental volumetric tomography

**DOI:** 10.4317/jced.61240

**Published:** 2024-02-01

**Authors:** Ridvan Guler, Kamil-Serkan Agacayak

**Affiliations:** 1 DDS, MSC, Dicle University, Faculty of Dentistry, Department of Oral and Maxillofacial Surgery, Diyarbakir, Turkey; 2 DDS, PHD, Dicle University, Faculty of Dentistry, Department of Oral and Maxillofacial Surgery, Diyarbakir, Turkey

## Abstract

**Background:**

HT is a systemic disease that presents with persistent high blood pressure, which has become an important health problem due to its cause of serious complications and high prevalence in the community. Aim: This study aims to examine the bone mineral density (BMD) of male patients using different groups of antihypertensive drugs for long terms with dental volumetric tomography.

**Material and Methods:**

The study was carried out using the data of patients who applied to the Dicle University Faculty of Dentistry and underwent the Dental Volumetric Tomography (DVT) scan for any reason. The patients included in the study were divided into 4 groups according to their antihypertensive use; Group 1: 60 patients who never used hypertensive medication before, Group 2: 60 patients who received Calcium Channel Blocker treatment for over 5 years, Group 3: 60 patients who received Beta Blocker treatment for over 5 years, Group 4: 60 patients who received ACE inhibitor treatment for over 5 years. Radiomorphometric measurements were made on the DVT data and the DVT-Mandibular Index Inferior, DVT-Cortical Index, Hounsfield Unit-Cortical and Hounsfield Unit-Spongios values were calculated. The Kruskal-Wallis test, the Mann Whitney test with Bonferroni correction, the One-Way ANOVA and Post-Hoc Tukey test were used in the study.

**Results:**

A significant increase in DVT-CI and a significant decrease in HU-CORTIKAL, HU-SPONGIOS and DVT-MII values were observed in the patients using Calcium Channel Blocker medication. These findings pointed to osteoporosis. In addition, no statistically significant difference in the use of antihypertensive drugs in the Beta Blocker and ACE Inhibitor groups compared to the Control Group were found.

**Conclusions:**

The long term use of Calcium Channel Blocker group antihypertensive drugs should be considered as a risk factor for osteoporosis in men.

** Key words:**Antihypertensive drug, Osteoporosis, Radiomorphometric index, Mandible, Dental volumetric tomography, Bone mineral density.

## Introduction

Hypertension (HT) or high blood pressure is a disease described as the mean arterial blood pressure being above normal values (1). Drugs used in the treatment of HT are classified in 5 groups. These groups being; diuretics, sympatholytics or adrenergic nervous system antagonists, drugs that affect the renin-angiotensin system, drugs that act on vascular smooth muscle, and drugs which are currently under development (2). Osteoporosis is a condition in which the bone tissue becomes significantly fragile due to the extreme decrease in bone density (3). It is characterized by a parallel decrease in bone matrix and minerals (3,4).

In addition to the many side effects in patients using antihypertensive drugs, there is also a risk of osteopenia and osteoporosis with the long term usage of these drugs (5). Therefore, the usage of antihypertensive drugs for over two years as well as the ages of 50 and higher can be considered as risk factors that may affect bone density (5,6).

Bone Mineral Density (BMD) measurements today are made with densitometers (7). There are many methods used to measure BMD in different regions of the skeleton. Some of these methods are Dual Photon Absorptiometry (DPA), Single Photon Absorptiometry (SPA), Quantitative Computed Tomography (QCT), Dual Energy X-Ray Absorptiometry (DEXA) and Single Energy X-Ray Absorptiometry (SXA). In addition to these methods, qualitative and quantitative evaluations of the jaw bones can be made with advanced imaging methods such as Conventional Radiographs and Dental Volumetric Tomography (DVT) (7,8)

DVT is an increasingly common technology that can produce high-resolution three-dimensional images of the head and neck region with a short scanning time and greatly reduced radiation dose compared to conventional Computed Tomography (CT). DVT is used in many recent studies in BMD measurements (9-12). Although DEXA is the most reliable technique in BMD measurement today, it cannot be used widely because it is a difficult to access and costly diagnostic tool (13). Therefore, many radiomorphometric analysis indices have been developed as an alternative to DEXA to detect patients with low BMD (13,14).

The aim of this study is to evaluate the effects of regular antihypertensive drug use on the cortical and trabecular bones of the mandible by performing radiomorphometric measurements on DVT images.

## Material and Methods

This study was planned as a retrospective study which included patients diagnosed with hypertension using regular antihypertensive drugs. The study was approved by the Dicle University, Faculty of Medicine, Non-Interventional Clinical Research Ethics Committee with the protocol number 251 on 19.09.2018. Informed consent was signed by all patients before the study. The study included male patients aged 25-75 years who were diagnosed with HT at least 5 years prior to administration at the Dicle University Faculty of Dentistry and used regular antihypertensive drugs; who applied to Dicle University, Faculty of Dentistry, Oral and Maxillofacial Surgery Polyclinic with different complaints and received DVT due to various dental indications. The blood pressures of patients were considered as normal and sTable under drug treatment and drug exposure data are based on self-report of patients. DVT’s from patients were taken for routine patient clinical examination, not for bone mineral density investigation.

All DVT images were taken on the same device (I-CAT vision TM Imaging Science International, Hatfield, USA) and by the same x-ray technician. In order to provide standardization in the DVT imaging, reference points determined by the manufacturer on the device were strictly followed. In patient positioning, attention was paid to ensure that the vertical line formed on the device was parallel to the sagittal plane of the patient and that the horizontal line passed through the Frankfurt plane and was parallel to the ground. Acquisition parameters were adjusted to be 120 kVp, 18.54 mA, 8.9 seconds, with a voxel size of 0.3mm and an image area of 13cm length and 10cm width.

-The study was designed as 4 groups:

Control Group (N = 60) - Patients who did not have any systemic diseases or regular usage of medication.

Calcium Channel Blocker Group (N = 60) – Patients who use long-term (a minimum of 5 years) Calcium Channel Blocker group drugs.

Beta Blocker Group (N = 60) - Patients who use long-term (a minimum of 5 years) Beta Blocker group drugs.

ACE Inhibitor Group (N = 60) – Patients who use long-term (a minimum of 5 years) ACE inhibitor group drugs.

Female patients, patients with a history of any cancer, substance abuse (smoking, alcohol and other drugs), diabetes mellitus, Paget’s disease, history of periodontitis, vertical-horizontal bone loss, patients taking vitamin supplements, thyroid dysfunction, daily physical activity, osteoporosis and patients who had received chemotherapy or any type of treatment for osteoporosis were excluded from the study. In addition, patients with serious pathologies such as defects, tumors and cysts in the jaw region to be examined in the DVT sections were also excluded from the study.

-Dental Volumetric Tomography Procedure

The DVT images of all the patients included in the study were evaluated by the same expert with I-CAT vision software. As radiomorphometric indexes; Dental Volumetric Tomographic Mandibular Index Inferior (DVT-MII), Dental Volumetric Tomographic Cortical Index (DVT-CI), Hounsfield Unit - Cortical (HU-CORTIAL) and Hounsfield Unit - Spongios (HU-SPONGIOS) were used. The radiomorphometric indexes were measured on the sections taken from the right side of the patient in panoramic and implant mode of the DVT images.

-Measurement of Radiomorphometric Parameters

Hounsfield Unit (HU)

The following points were designated on a coronal cross section in implant mode:

● The site 3 mm lateral to the upper cortical line of the mental foramen with the inclusion of 1 mm2 of spongiosis bone (HU-SPONGIOSIS)

**Figure 1:**
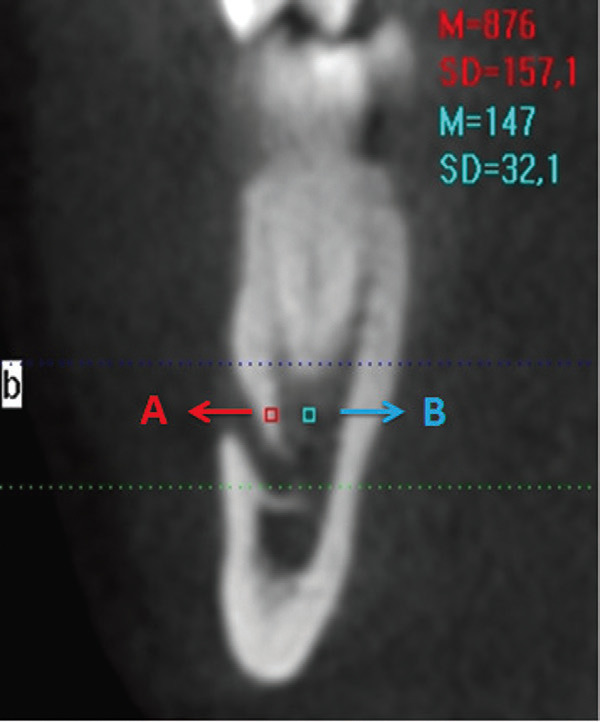
On coronal section in implant mode: A. HU SPONGIOSIS, B. HU CORTICAL.

● The site on the upper cortical wall of the mental foramen that included 1 mm2 of cortical bone (HU-CORTICAL). HU values of these sites were calculated (Fig. 1).

Evaluation of Hounsfield Units (HU)

Bone density in DVT images are expressed in Hounsfield Units (HU) (15). HU values range from -1000 to +3000. -1000 indicates the value of pure air and +3000 indicates the metal with the highest density. This evaluation indicates that water has the value of 0 HU and soft tissue has a value close to 0 or a negative value. However, bone tissue gives results between +100 and +1900 HU (16). The values corresponding to various HU values under the Misch classification are given below (10).

● D1 bone: >1250 HU

● D2 bone: 850–1250 HU

● D3 bone: 350 −850 HU

● D4 bone: 150–350 HU

● D5 bone: 0–150 HU

-Dental Volumetric Tomography - Mandibular Index Inferior (DVT-MII)

**Figure 2:**
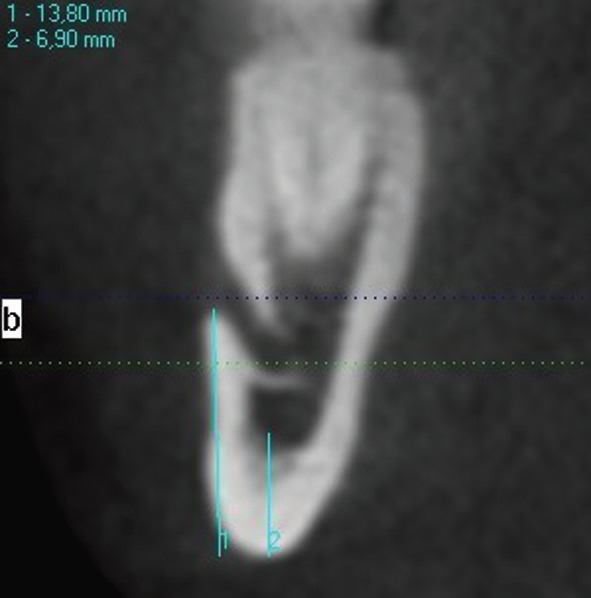
DVT-MI measurement: 1-MFU, 2-BMC.

In this method, the mental foramen region was used as a reference for BMD. The right mental foramen was detected by examining the coronal sections of the patients DVT images in implant mode. The distance of the line starting from the upper border of the mental foramen cortical bone to the lower border (MFU) and the distance parallel to this line and extending to the lower (inferior) border of the mental foramen (BMC) were measured and proportioned to each other (Fig. 2).

Dental Volumetric Tomography - Cortical Index (DVT-CI) 

**Figure 3:**
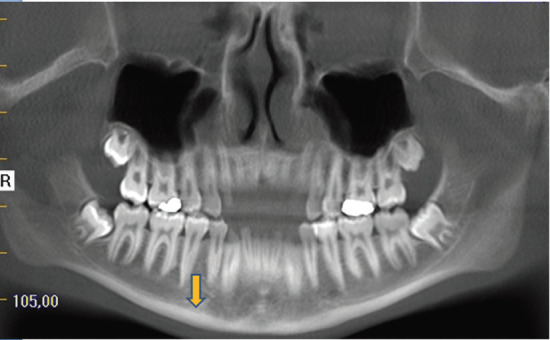
DVT-CI=1, integrity of the cortical bone.

**Figure 4:**
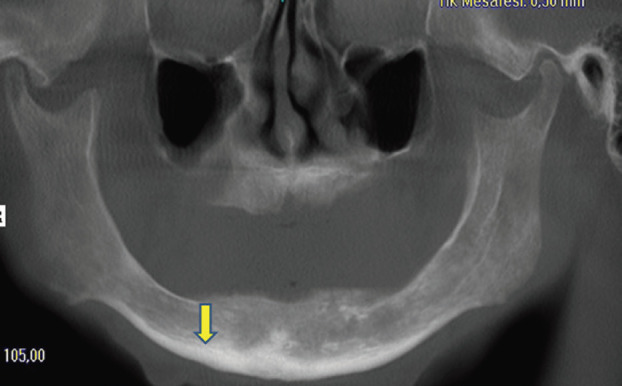
DVT-CI=2, cortical bone with resorbed cavities or residues of one to three sizes and small size.

• DVT-CI =1: Smooth cortical endosteal margin bilaterally (Fig. 3).

• DVT-CI =2: Resorption cavities and stratification (1-3 pieces) at the endosteal border uni/bilaterally (Fig. 4).

• DVT-CI =3: Distincly porous endosteal margin.

-Statistical Analyses

Statistical analyses were performed using the SPSS (Statistical Package for Social Science) version 15.0 existing in the computer package program. In order to determine statistically significant differences in the variables of the four groups, the normal distribution of these variables were examined. The One-Way ANOVA and Post-Hok Tukey tests were used to compare the four groups in terms of normally distributed variables (*p*<0.05). The Kruskal-Wallis test was used for the triple comparison of the groups for variables that did not show normal distribution (p≤0.008). The Mann Whitney test with Bonferroni correction was used for the pairwise comparison of the groups showing a significant difference (p≤0.008).

## Results

Male patients aged 25-75 years with a mean age of 58.7±7.1 were included in the study. The statistical properties of the radiographic parameters of all patients included in the study are shown in Table (Table 1). According to Table 1, the DVT-MII mean of all patients included in the study was 0.36±0.05, the DVT-CI mean was 1.55±0.65, the HU-SPONGIOS mean was 324.3±97.6, and the HU-CORTICAL mean was 724.4±77.9. According to the results of the study, a statistically significant difference was found in the DVT-MII parameter between the Control group and the 3 groups with antihypertensive drug use (*p*<0.05). In the HU-CORTICAL parameter, there was a statistically significant difference between the Control group and the Ca Channel Blocker group (*p*<0.05) while there was no statistically significant difference between the Control group and the Beta Blocker and ACE Inhibitor groups (*p*>0.05) (Table 2).

**Table 1:**
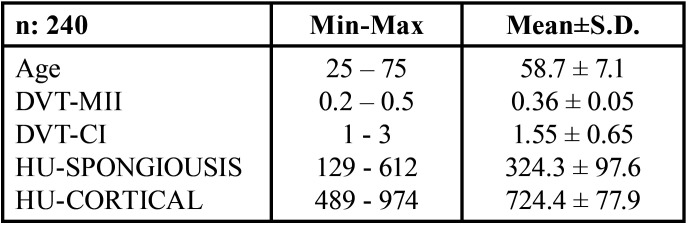
Evaluation of the radiographic parameters in the patients. Abbreviations: DVT-MII, Dental Volumetric Tomography - Mandibular Index; DVT-CI, Dental Volumetric Tomography – Cortical Index; HU, Hounsfield Units.

**Table 2:**
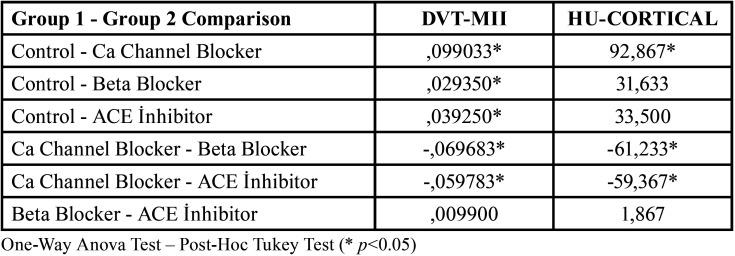
Comparison of the mean of DVT-MII and HU-CORTIAL parameters of all groups.

In addition, while there was a statistically significant difference in the DVT-CI parameter between the Control group and the group using Ca Channel Blocker medication (*p*<0.008), there was no significant difference between the Control group and both the Beta Blocker and ACE Inhibitor groups. In the HU-SPONGIOSIS parameter, there was a statistically significant difference between the Control group and the Ca Channel Blocker group (*p*<0.008), while there was no statistically significant difference between the Control group and both the Beta Blocker and ACE Inhibitor groups (Table 3).

**Table 3:**
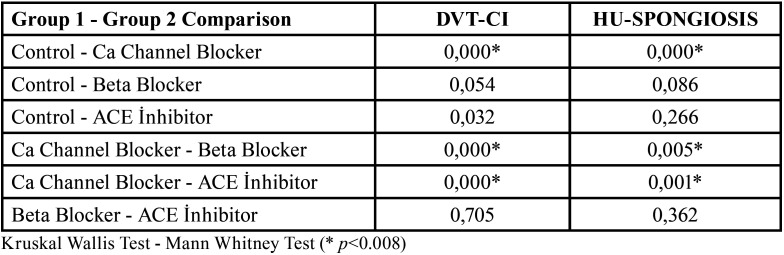
Comparison of the mean of DVT-CI and HU-SPONGIOSIS parameters of all groups.

In the comparative statistical analysis between 3 groups with antihypertensive drug use, there was a statistically significant difference between the Ca Channel Blocker and Beta Blocker and Ca Channel Blocker and ACE Inhibitor groups according to all parameters (DVT-MIII, DVT-CI, HU-SPONGIOS, HU-CORTICAL). There was no significant difference between the Beta Blocker and ACE Inhibitor groups ( Table 2, Table 3). As a result; a significant increase in DVT-CI and a significant decrease in HU-CORTIAL, HU-SPONGIOS and DVT-MII values were observed in patients using Calcium Channel Blocker medication.

## Discussion

The average life expectancy of the human population is gradually increasing in accordance to the level of development in countries around the world. The prolongation of life expectancy leads to an increase in chronic diseases. In addition; socioeconomic levels, cultural habits, change of lifestyle, stress and inactivity are other factors that affect the increase in chronic diseases of individuals (17). Since osteoporosis is an extremely important health problem especially in the elderly due to the increased risk of bone fractures; the difference between the three drug groups compared in this study, which are frequently used in hypertensive patients, are of great importance (18).

In addition, osteoporosis and hypertension are seen together in many patients today. Regular and long-term use of antihypertensive drugs may lead to potential adverse effects on the bone tissue in these patients. Many antihypertensive drugs have been associated with osteoporosis and fractures (19). However, it is unclear whether antihypertensive drugs affect bone tissue directly or indirectly. Epidemiological studies report that some antihypertensive drugs such as thiazide diuretics and beta-blockers reduce the risk of bone fractures, especially in postmenopausal women. The effects of other antihypertensive drugs on bone tissue, however, are controversial (10,18,19).

The increase in BMD in patients using thiazide group drugs is attributed to the decrease in urinary calcium excretion and the drugs effect on intracellular calcium regulation (20). The effects of beta-blockers on BMD have also been investigated in a number of previous studies (10,21) These studies have shown that the inactivation of the sympathetic nervous system increases bone formation by impairing the osteoclastic bone resorption in animal models (10,21). It was determined that receptor activation of leptin and nuclear factor IB ligand played a role in this relationship.

In general, the effects of these drugs on bone mineral metabolism are multifactorial and the mechanism has not been clearly explained. There are also many studies on this subject (10,22-25)

In a study conducted in 2007, Bonnet *et al*. (22) examined the BMD effect of beta-blocker antihypertensive drugs in postmenopausal women. The bone geometry of the femoral neck in DEXA images were examined and it was reported that long term use of beta-blockers were associated with a lower bone fracture risk (22). Barzilay *et al*. (23) studied the effects of antihypertensive drugs on BMD and bone fracture risk. As a result of the study, it was reported that thiazide diuretics have a more protective effect against bone fractures compared to Ca channel blockers and beta blockers.

Pasco *et al*. (24) investigated the effect of beta-adrenergic blockers on BMD. As a result of the study, it was reported that beta-blockers were associated with a decrease in the bone fracture risk and an increase in BMD. While a decrease in bone density and increase in bone fracture risk was observed in the Ca Channel Blocker Group in the current study, the absence of a significant difference in BMD between the Beta Blocker Group and the Control Group supports this study.

Lynn *et al*. (25) investigated the relationship between ACE inhibitor usage and BMD in 3887 Chinese individuals (1958 men and 1929 women). As a result of this study, a higher BMD was reported in Chinese individuals using ACE inhibitors.

Ağaçayak *et al*. (10) investigated the effects of long-term antihypertensive treatment with Ca channel blockers and beta blockers on mineral density in the maxillary bone. As a result, it was reported that beta blockers could be preferred to Ca channel blockers in the treatment of hypertension in patients with a high risk of osteoporosis and bone resorption. The data obtained from the current study shows parallelism with the literature.

There is still no standardized method or standardized values for the evaluation of osteoporosis with DVT. Further studies are warranted to specify standardized values. If a standardization was established, patients who had to have a DVT with dental indications might not need to have an extra dual-energy X-ray absorptiometry measurement for the evaluation of osteoporosis, and in this way, one DVT may serve two purposes.

## Conclusions

It is exceedingly important for dentists to be aware of the side effects of antihypertensive drugs in order to prevent the risk of osteoporosis and osteopenia in hypertensive patients. Dentists have a particularly important role in the selection and dose adjustment of the appropriate drugs for hypertensive patients at risk of osteoporosis. With this study, it can be determined that beta blockers and ACE inhibitors can be preferred to Ca channel blockers in the treatment of hypertension in patients with a high risk of osteoporosis.
